# Changing the Vision in Smart Food Design Utilizing the Next Generation of Nanometric Delivery Systems for Bioactive Compounds

**DOI:** 10.3390/foods9081100

**Published:** 2020-08-12

**Authors:** Francesco Donsì, Giovanna Ferrari

**Affiliations:** 1 Department of Industrial Engineering, University of Salerno, via Giovanni Paolo II, 132, 84084 Fisciano, Italy; gferrari@unisa.it; 2 ProdAl Scarl, via Giovanni Paolo II, 132, 84084 Fisciano, Italy

**Keywords:** bioactive compounds, delivery systems, nanotechnology, bioavailability, gut microbiota, toxicity

## Abstract

In modern foods, the delivery systems for bioactive compounds play a fundamental role in health promotion, wellbeing, and disease prevention through diet. Nanotechnology has secured a fundamental role in the fabrication of delivery systems with the capability of modulating the in-product and in-body behavior for augmenting bioavailability and activity of bioactive compounds. Structured nanoemulsions and nanoparticles, liposomes, and niosomes can be designed to improve bioactives preservation after ingestion, mucoadhesion, as well as of their release and pathophysiological relevance. In the future, it is expected that the delivery systems will also contribute to augment the efficacy of the bioactive compounds, for example by improving the intestinal absorption and delivery in the bloodstream, as well as promoting the formation of additional bioactive metabolites by regulating the transformations taking place during digestion and the interaction with the intestinal microbiota.

The growing demand for health promotion, wellbeing and disease prevention through diet and nutrition has stimulated the development of smart foods, characterized by advanced functionality for the delivery to the target sites of the bioactive compounds they contain. Until recently, the main challenges to the incorporation of bioactive compounds, such as phytochemicals, micronutrients, and dietary fibers, in foods were related to overcoming the issues of poor dispersibility in the food matrix, unpleasant taste and physicochemical instability under food processing conditions (exposition to high temperatures, light, oxygen, high shearing, as well as interaction with food matrix ingredients) [1,2,3]. However, the driving force towards smart foods has caused the emergence of the need for enhanced health benefits through the triggered, targeted and controlled release of the bioactive compounds and augmented bioavailability in the gastrointestinal tract, to overcome the issues of insufficient gastric residence time and low permeability within the gut [2,4,5].

The food industry has extensively relied on the use of nanotechnology, as an essential tool to extend the capability of controlling the in-product and in-body behavior of delivery systems for bioactive compounds, with applications in agriculture, foodstuffs transformation, packaging, and storage, and the production of dietary supplements [6,7]. Coherently with the need for cost-effective and clean-label products, applications have mainly focused on relatively simple systems, such as colloidal emulsions [3], micelle [8], and biopolymeric nanoparticles [9].

Recently, Martínez-Ballesta et al. have reviewed the use of novel nanosized encapsulation and delivery systems to expand the field of application of bio-functional nutraceutical compounds [10], which can be projected to find use in the “smart food for the health” in the near future. More specifically, the control of bioactives preservation after ingestion, mucoadhesion, as well as of their release and pathophysiological relevance requires more advanced delivery systems, such as structured nanoemulsions and nanoparticles, liposomes, and niosomes [10,11]. The structural complexity of the core and interfacial layer of these systems contributes, in conjunction with their nanometric size and composition, to the development of unique functionalities [12].

Nanoemulsions offer several inherent advantages for the food industry, especially the simplicity of production, high loading capability, high stability against creaming, and optical transparency [4,13], but lack of control over the release of the encapsulated bioactive compounds. Structural complexity for more advanced functionality can be introduced in the nanoemulsion core or shell. For example, nanoemulsions with the core made of crystallized biocompatible lipids, also known as solid-lipid nanoparticles (SLNs) [14], contribute to improving the preservation of the payload from degradation, enabling controlled release, targeted delivery [15], and enhanced bioavailability [16]. Nanostructured lipid carriers (NLCs), made of partially crystallized lipid droplets, are considered the next generation of SLNs because the less ordered crystalline or totally amorphous structure of their core promotes both particle stability and loading capacity and enables tailored release properties [16,17]. Nanoemulsions with a structured interface, achieved with the deposition of multiple layers of emulsifiers and/or polyelectrolytes through electrostatic and hydrophobic interactions [18], exhibit greatly improved physical stability under extreme conditions of temperature, pH and ionic strength [19], by increasing the packing density at the interface [20], enhancing absorption properties in the gastrointestinal tract [21], controlling or triggering release upon using functionalized polymers [22], and responding to external stimuli [21,23]. Liposomes are nanosized artificial vesicles, typically made of bilayers of phospholipid and cholesterol, which have found wide use as immunological adjuvants and drug carriers [24,25]. They are characterized by the capability to encapsulate both hydrophilic and hydrophobic molecules, high structural versatility, and capability to augment the biological activity of the encapsulated compounds, but limited delivery in vascularized tissue because of their scarce stability in plasma [10]. Hybrid nanoparticles, made of liposomes coated with polyelectrolytes by electrostatic interaction, have been reported to markedly improve liposomal stability, both in the food product and during digestion [26]. Niosomes, which are structurally similar to liposomes, represent an effective alternative to these carriers. They are formed by a non-ionic surfactant of alkyl or dialkyl polyglycerol ether class and cholesterol [27], and, because of their non-ionic nature, they are less toxic, while providing an improved therapeutic index for bioactive compounds [28]. In the case of biopolymeric nanoparticles, more advanced functionalities are pursued mainly through the exploitation of the functional properties of macromolecules, such as polysaccharides and proteins, alone or in combination, which are able to self-assemble, aggregate or interact with other molecules as a function of the environmental conditions, and to respond in a controlled way during gastrointestinal digestion and blood circulation [9,29]. Natural polysaccharides, including starch, cellulose, pectin, guar gum, chitosan, alginate, cyclodextrins, native gums, and their combinations and chemically modified forms, are promising inexpensive delivery systems for bioactive compounds. Because of their large molecular structure, with different reactive groups and hydrophobic and hydrophilic moieties, they are suitable for the effective entrapment of different bioactive compounds [29]. Food proteins, both of animal (gelatin, dairy and egg proteins) and plant (soy proteins, pulses proteins, and cereal proteins) origin [30], exhibit high binding ability to hydrophobic and hydrophilic small molecules [9,31], surface activity, self-assembling properties, and good gelation characteristics [9].

Although biocompatible particles are generally considered as safe for human consumption [2], the risks associated with toxic effects deriving from long-term use and overdoses due to nonspecific toxicity have to be carefully assessed [32,33]. Additionally, there are major concerns about how the inclusion of nanomaterials in food formulation or packaging could compromise food safety [10,34], for example reaching tissues naturally protected by biological barriers, such as the blood–brain barrier [35,36]. Furthermore, depending on the nature of nanoparticles and the surrounding environment, major changes could take place, through aggregation, modification of chemical properties, size, and shape [37], modifying the toxicological profile of the delivery systems. In this respect, current legislation, which is mostly limited to the requirement of labeling food products containing nanomaterials, does not provide an adequate regulatory frame for the use of nanotechnologies in foods [10]. In contrast, nanomaterials toxicity should be assessed and regulated with reference to the food matrix, where they are incorporated [38].

Because of the lack of knowledge on potential risks, Martínez-Ballesta et al. suggest that the next generation of delivery systems for bioactive compounds should focus on enhancing the bioavailability and activity of the bioactive compounds, to reduce dosages [10]. This can be achieved in the first place by improving the performance of the delivery systems; for example, by promoting mucoadhesiveness and the capacity to open tight junctions, improving paracellular transport [39,40], enhancing transmembrane transport efficiency [41], or, once absorbed from the intestinal lumen, modulating the exposition to metabolizing enzymes in enterocytes [42], controlling the length of circulation in the bloodstream [43], modifying the pharmacokinetics of bioactive phytochemicals [10], and achieving site-specific delivery, possibly accompanied by sustained therapeutic effects [44]. Moreover, the next generation of delivery systems should also take advantage of the transformations taking place during digestion and the interaction with the intestinal microbiota, for producing additional bioactive metabolites that are able to create a synergistic health-beneficial effect with the administered bioactive compounds [41,45]. Finally, it is expected that the production of the next generation of delivery systems is not only economically viable for the food industry, but is also carried out according to the principles of green chemistry, using eco-friendly and biocompatible reagents and processes, which avoid organic solvents and use natural biomaterials (such as plant extracts and microorganisms) [46], hence minimizing the environmental impact [10], and protecting resources and human health [47].
